# Professional Status of Persistent Spinal Pain Syndrome Patients after Spinal Surgery (PSPS-T2): What Really Matters? A Prospective Study Introducing the Concept of “Adapted Professional Activity” Inferred from Clinical, Psychological and Social Influence

**DOI:** 10.3390/jcm10215055

**Published:** 2021-10-29

**Authors:** Nicolas Naiditch, Maxime Billot, Lisa Goudman, Philippe Cornet, Manuel Roulaud, Amine Ounajim, Philippe Page, Bertille Lorgeoux, Sandrine Baron, Kevin Nivole, Pierre Pries, Yassine Abdollah Moufid, Cécile Swennen, Simon Teyssedou, Tanguy Vendeuvre, Elodie Charrier, Laure Poupin, Delphine Rannou, Géraldine Brumauld de Montgazon, Pierre François Descoins, Brigitte Roy-Moreau, Nelly Grimaud, Romain David, Maarten Moens, Philippe Rigoard

**Affiliations:** 1 Prismatics Lab., Predictive Research in Spine/Neuromodulation Management and Thoracic Innovation/Cardiac Surgery, Poitiers University Hospital, 86021 Poitiers, France; Manuel.ROULAUD@chu-poitiers.fr (M.R.); Amine.OUNAJIM@chu-poitiers.fr (A.O.); Bertille.LORGEOUX@chu-poitiers.fr (B.L.); sandrine.baron@chu-poitiers.fr (S.B.); Kevin.NIVOLE@chu-poitiers.fr (K.N.); romain-david@hotmail.fr (R.D.); philippe.rigoard@chu-poitiers.fr (P.R.); 2 Euridol, Neuropôle de Strasbourg, Faculty of Life Science, University of Strasbourg, 67000 Strasbourg, France; 3 Department of Neurosurgery, Universitair Ziekenhuis Brussel, 1090 Brussels, Belgium; lisa.goudman@gmail.com (L.G.); mtmoens@gmail.com (M.M.); 4 Stimulus Research Group, Vrije Universiteit Brussel, 1090 Brussels, Belgium; 5 Department of General Medicine, Sorbonne University, 75012 Paris, France; cornetphilippe@hotmail.com; 6 Department of Spine Surgery & Neuromodulation, Poitiers University Hospital, 86021 Poitiers, France; Philippe.PAGE@chu-poitiers.fr (P.P.); Pierre.PRIES@chu-poitiers.fr (P.P.); Abdollah-Yassine.MOUFID@chu-poitiers.fr (Y.A.M.); Cecile.SWENNEN@chu-poitiers.fr (C.S.); Simon.TEYSSEDOU@chu-poitiers.fr (S.T.); tanguy.vendeuvre@chu-poitiers.fr (T.V.); 7 Pain Evaluation and Treatment Centre, Poitiers University Hospital, 86021 Poitiers, France; Elodie.CHARRIER@chu-poitiers.fr (E.C.); laure.poupin@chu-poitiers.fr (L.P.); delphine.rannou@chu-poitiers.fr (D.R.); 8 Pain Evaluation and Treatment Centre, La Rochelle Hospital, 17000 La Rochelle, France; Geraldine.DEMONTGAZON@ght-atlantique17.fr; 9 Pain Evaluation and Treatment Centre, Nord Deux-Sèvres Hospital, 79000 Niort, France; pierre-francois.descoins@ch-niort.fr (P.F.D.); Roy-Moreau.Brigitte@chnds.fr (B.R.-M.); 10 Pain Evaluation and Treatment Centre, Centre Clinical Elsan, 16800 Soyaux, France; ngrimaud@centre-clinical.fr; 11 Physical and Rehabilitation Medicine Unit, Poitiers University Hospital, University of Poitiers, 86021 Poitiers, France; 12 Pprime Institute UPR 3346, CNRS, ISAE-ENSMA, University of Poitiers, 86360 Chasseneuil-du-Poitou, France

**Keywords:** professional status, social factors, social gradient, Failed Back Surgery Syndrome, unemployment, chronic pain, social workers, inference in medicine

## Abstract

Persistent Spinal Pain Syndrome Type 2 (PSPS-T2) represents a main cause of work disruption. Beyond its societal consequences, occupational inactivity is responsible for a major decrease in physical/mental health in individuals but remains poorly analyzed. We designed a study to prospectively examine Professional Status (PS) evolution and its association with key bio-psychological markers. Data from 151 consecutively included working-age PSPS-T2 patients were analyzed to determine the proportion of professional inactivity and the relationships between PS and Social Gradient of Health (SGH), Numeric Pain Rating Scale (NPRS), EuroQol 5-Dimensional 5-Level (EQ-5D-5L), Oswestry Disability Index (ODI), Hospital Anxiety and Depression Scale (HADS), and Fear-Avoidance Belief Questionnaire work subscale (FABQ-W). Despite optimized medical management, 73.5% of PSPS-T2 patients remained inactive after 1 year of follow-up/*p* = 0.18. Inactive patients presented a low SGH/*p* = 0.002, higher NPRS/*p* = 0.048, lower EQ-5D-5L/*p* < 0.001, higher ODI/*p* = 0.018, higher HADS-D/*p* = 0.019 and higher FABQ-W/*p* < 0.001. No significant mediation effect of FABQ-W on SGH consequences regarding PS was observed in our structural model/*p* = 0.057. The link between unemployment and bio-psycho-social pain dimensions appears bidirectional and justifies intense collaboration with social workers. Optimizing therapeutical sequencing towards personalized professional plans implies restoring “Adapted Physical Function” as an initial goal, and tailoring an “Adapted Professional Activity”, matching with patient expectations and capabilities, as a final objective.

## 1. Introduction

Low back pain is a huge worldwide problem affecting more than 30% of global population [1]. While resolutive evolution can be observed for 80–90% in 3 months, the remaining population falls into the category of chronic pain [1]. In case of failure, Patient Optimized Medical Management (OMM) can lead to spinal surgery designed to address mechanical features with variable outcomes [2,3]. Despite surgery, patients can develop new onset of pain or experience persistence of their back and/or leg pain, defining Persistent Spinal Pain Syndrome Type 2 (PSPS-T2) or Failed Back Surgery Syndrome (FBSS) [4,5]. PSPS-T2 represents one of the most challenging and disabling conditions, with prevalence estimated from 10% to 50% [4,6,7], dramatically impacting quality of life [8]. While it has been well-documented that PSPS-T2 affects biological and psychological factors, very few studies have investigated its societal consequences by focusing on social factors [9,10,11,12]. By assessing the Social Gradient of Health (SGH) (relationship between health and socioeconomic position) in a prospective real-life study, we recently demonstrated that PSPS-T2 patients with low SGH represented more than 85% of PSPS-T2 patients [12]. The unemployment rate has been estimated between 50.9% to 81.5% for PSPS-T2 patients [13,14,15,16,17], generating a huge financial burden [18]. Cessation of professional activity has been shown to have a dramatically negative effect on well-being and health [19,20,21], reinforcing the detrimental effect of PSPS-T2 on quality of life [22].

Given this context, emphasis has been laid on pain intensity management, using a systematic pharmacological approach and psychological distress evaluation, in order to rehabilitate patients in their previous professional activity [23]. However, we must recognize that OMM leaves limited space for social management, since social workers nowadays remain poorly represented in the MultiDisciplinary Team (MDT) algorithm of the vast majority of pain management structures.

Focusing on psychological distress associated with chronic pain, numerous studies have reported significant correlation between kinesiophobia associated with work (i.e., fear of movement) and number of missed work days [14,24,25,26]. Fear of movement detrimental to productivity can ultimately lead to unemployment [26]. In addition to fear of movement, physical incapacity related to workplace drudgery might be considered as a true limitation to return to work, above and beyond pain intensity and the burden of psychological kinesiophobia [27,28,29,30]. Although the economic impact of pain-related work disability is undeniable, Professional Status (PS) is still today considered as a global concept, without any comprehensive view of this specific condition, from the nature of work arduousness to the psychological and social parameter evaluation of patients suffering from PSPS-T2. Nevertheless, we can assume that consideration of these aspects might have substantial implications on patient pathway therapeutic management.

Aiming to evaluate social factor consequences on Professional Status in PSPS-T2 patients, we have designed a prospective observational study including 200 PSPS-T2 patients. The primary objective was to determine the proportion of professional inactivity in the working-age sub-population. The secondary objectives were to examine (i) kinesiophobia association with work (FABQ-W) and PS, (ii) the correlation between the SGH and PS, and (iii) evolution of PS after 12 months of “optimal” care. Our ambition was also to lay stress on return-to-work difficulties, due to kinesiophobia (psychological aspects) and/or to the arduousness of work (social aspects), focusing on pain pathway management related to functional capacity.

## 2. Materials and Methods

### 2.1. Study Design

This prospective observational multicenter PREDIBACK study enrolled 200 PSPS-T2 patients from January 2017 to March 2018 in 5 French pain clinics (Poitiers, Bressuire, Niort, La Rochelle and Angoulême). The PREDIBACK study aimed to characterize PSPS-T2 patients through clinical, psychological, and social assessment (https://clinicaltrials.gov/ct2/show/NCT02964130; Last accessed: 23 October 2021). The PREDIBACK study was approved by the Ethics Committee (CPP Ouest III) and the ANSM (2016-A01144-47). All patients provided their informed consent before enrolment.

### 2.2. Patient Selection

Inclusion criteria included patients aged between 18 and 80 years who had undergone at least one spinal surgery, experienced post-operative back and/or leg pain for at least 6 months despite surgery and were suffering from an average global pain score ≥ 4 on the NPRS [31]. 

Patients who had previously been implanted with neurostimulation treatment (Spinal Cord Stimulation, subcutaneous or peripheral nerve stimulation), intrathecal drug delivery; had life expectancy less than 1 year; had not the physical or intellectual capacity to endure study assessments or to perform survey alone; were members of a vulnerable population; or were suspected of misusing that could bias the study results were excluded from this study screening log.

### 2.3. Outcome Measurements

#### 2.3.1. Social Outcomes

The professional situation was declaratively assessed on a way according to 5 modalities: active [32], disabled [33], sick leave [34], long-term sick leave [35], and unemployed [36]. Disability, long-term sick leave, sick leave and unemployed responses were aggregated under the name “inactive”. Analysis focused on working-age patients and retired were excluded from the analysis. 

Profession and Socio-Professional Category (PSC) was used to determine the social gradient: Farmers (PSC 1), craftsmen, salesmen and managers (PSC 2), blue-collar workers (PSC 6) and lower-grade white-collar workers (PSC 5) were considered as “Low Social Gradient of Health (SGH)” patients. Technicians and associate Professionals (PSC 4) and Professionals (PSC 3) were considered as “High Social Gradient of Health (SGH+)” patients.

Educational level was collected according to two categories: “<upper secondary education” and “≥upper secondary education”. Upper secondary education was set as a cut-off regarding its suitability in PS evaluation [12]. 

#### 2.3.2. Clinical Outcomes

Global pain intensity over the past 5 days was assessed with a Numeric Pain Rating Scale (NPRS) [37] (0 = no pain to 10 = the worst imaginable pain). 

Functional disability was assessed with the Oswestry Disability Index (ODI) [38,39] consisting in 10 items (0 = No disability to 50 = total disability).

Quality of life was assessed by EuroQol 5-Dimensional 5-Level questionnaire (EQ-5D-5L) (0 = worse than dead to 1 = full health) [40,41].

#### 2.3.3. Psychological Outcomes

Kinesiophobia, equated with fear of movement, was evaluated by the Fear-Avoidance Belief Questionnaire (FABQ) [42,43,44], consisting in 16 items split into 2 subscales. The first subscale, FABQ-Physical Activity (FABQ-PA), measured kinesiophobia related with physical activity (5 items) and the second subscale, FABQ-Work (FABQ-W), measured kinesiophobia related with work (11 items). The maximum score was 24 for the FABQ-PA and 42 for the FABQ-W. 

Psychological distress was assessed by using the Hospital Anxiety and Depression Scale (HADS) [45,46] consisting in 14 items: 7 for the anxiety subscale (HADS-A) and 7 for the depression subscale (HADS-D). Scores range from 0 to 21.

### 2.4. Statistical Analysis 

Mean and standard deviation (SD) were used to describe quantitative variables. Frequency and percentages (%) were used to describe qualitative variables. The Shapiro-Wilk test was used to assess the data normality distribution.

The bivariate relationships between professional status (PS) and each of the variables: Social Gradient of Health (SGH), educational level and sex, were analyzed with the Chi^2^ test. Relationships between PS and NPRS, ODI, EQ-5D-5L, HAD and FABQ-W were evaluated using the Mann-Whitney-Wilcoxon tests (no variable followed a normal distribution).

Patient changes in PS between baseline and one year of follow-up were analyzed using a Cochrane Q-test. The analysis was performed to assess changes in employment status between the inclusion visit and the one-year follow-up visit, focusing on working-age patients, who were normally active and had completed both the initial and final visits.

Logistic regression, with PS (active/inactive) as output and SGH and FABQ-W as input, was used to estimate the adjusted effects of SGH and FABQ-W on PS. Mediation effect analysis was performed to evaluate the mediation effect of FABQ-W scores on the global effect of SGH on PS (i.e., does work-related kinesiophobia explain the SGH differences in professional status?). Logistic regression, standardized coefficients and their standard errors were reported. As our response variable was binary, we conducted the mediation analysis using structural equation modeling with the *sem* function available in the *lavaan* package [47] of R software to compute the indirect effect and its standard error and *p*-value. Coefficients in the structural equation model were estimated using the diagonally weighted least squares and standard errors were estimated using bootstraps.

All statistical analyses were conducted as available-case analyses based on completed assessments.

The R software was used to perform statistical analyses (R Development Core Team, Vienna, Austria, 2010). *p*-values of <0.05 were considered significant. 

## 3. Results

### 3.1. Occupational Status of Patients in the PREDIBACK Study

Results of professional work status are presented in Table 1. Out of the 200 enrolled patients, 19% (38/200) were retired and PS was unavailable for 5.5% of them (11/200). After exclusion of these patients, analysis was performed on a sample of 151 PSPS-T2 patients. 

Working-age employed patients represented 26.5% (40/151) of the total sample, and 73.5% of our study population (111/151) were inactive. Among inactive patients, 26.5% (40/151) were on sick leave, 25.2% (38/151) were on disability, 10.6% (16/151) were on long-term sick leave and 11.3% (17/151) were active unemployed. 

### 3.2. Patient Social Characteristics According to Professional Situation 

Patient social characteristics according to their professional situation are available in Table 2. 

The mean age and gender were not different according to professional situation (*p* = 0.542 and *p* = 0.824, respectively). Inactive patients had a significantly lower SGH than active patients (−19.4 points, *p* = 0.002). Inactive patients tended to be more numerous to have educational level lower than upper secondary education, in comparison with active patients, but the result was not significant (−13 points, *p* = 0.071).

### 3.3. Association between the Professional Situation and Medical Assessment

Results are available in Table 3.

The average pain intensity (NRPS) was significantly higher for inactive than for active patients (+0.5, *p* = 0.048). The mean functional disability (ODI) score was significantly higher for inactive patients than for active patients (+6.4 points, *p* = 0.018). The mean quality of life score was significantly lower for inactive than for active patients (−0.16, *p* < 0.001). 

### 3.4. Association between Professional Situation and Psychological Characteristics

Results of association between professional situation and psychological characteristics are presented in Table 4.

The mean score of Fear-Avoidance Belief Questionnaire work subscale (FABQ-W) was significantly higher for inactive than for active patients (+11.1, *p* < 0.001). However, the Fear-Avoidance Belief Questionnaire physical activity subscale (FABQ-PA) was not significantly different according to professional situation (*p* = 0.225). The mean score of depression (HADS-D) was significantly higher for inactive than for active patients (+1.8, *p* =0.019). Professional situation had no significant effect on the anxiety score (HADS-A, *p* = 0.117).

### 3.5. Professional Status, Social Gradient of Health and Kinesiophobia Associated with Work

**Figure 1:**
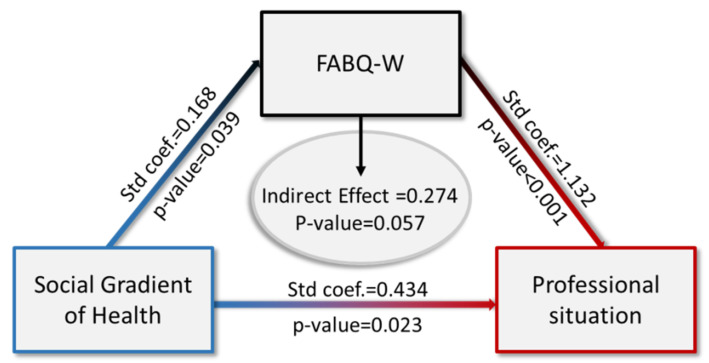
Structural equation model of the mediation effect of the Fear-Avoidance Belief Questionnaire Work subscale score on the effect of Social Gradient of Health on professional situation.

Results of Professional status, SGH and Kinesiophobia associated with Work are presented in Figure 1. 

Inactive patients were more likely to be SGH- than SGH+ (coef. = 0.496; 95% CI (0.165; 0.827), *p* = 0.003). The potential FABQ-W mediation effect related to the effect of SGH on professional situation was analyzed by our logistic regression model, which showed that inactive patients were more likely to be SGH- than SGH+ (coef. = 0.434; 95% CI (0.060; 0.808), *p* = 0.023) when considering FABQ-W scores. Patients with high kinesiophobia associated with work were significantly more inactive than active (coef. = 1.153; 95% CI (0.727; 1.579), *p* < 0.0001). FABQ-W score did not have a moderating effect on professional situation (*p* = 0.057). We found a significant effect of SGH on FABQ-W scores (coef. = −0.168; 95% CI (−0.010; 0.326), *p* = 0.039) and on professional situation (coef. = 0.434; *p* = 0.023). In our structural equation model, we did not find any significant mediation effect of FABQ-W scores on the effect of SGH regarding professional situation (indirect effect of 0.274; CI95% (−0.010; 0.558), *p* = 0.057). 

### 3.6. Evolution of Patients’ Professional Situations

Results of the professional situation evolution are presented in Table 5.

At 12 months, the professional situation of the 106 patients who completed both initial and 12-month visit was not significantly modified (*p* = 0.18), whereas 19% (20/106) of them changed their employment status. The number of active patients increased by 6.5% (7/106) from baseline to 12 months. The professional situation of the 30 patients on sick leave at the initial visit had changed significantly at 12 months (*p* = 0.01) with a proportion of 30% (9/30) returning to work, 10% (3/30) were recognized as being disabled or on long-term sick leave, and 3.3% (1/30) became unemployed.

## 4. Discussion

This study documents that more than 2/3 of PSPS-T2 patients were professionally inactive at the moment of their diagnosis of Persistent Spinal Pain Syndrome after spinal surgery. The majority of these patients had low SGH and a low educational level compared to the professionally active PSPS-T2 patients, which is not a classical notion shared in the medical community. 

While the SGH and kinesiophobia associated with work were significantly correlated with PS, our structural equation model was unable to show a significant mediation effect of the Fear-Avoidance Belief Questionnaire Work score on the effect of SGH regarding professional situation. Finally, after a 12-month period of follow-up, no significant professional situation changes were observed despite “Optimized Medical Management”. 

In light of the influence of clinical, psychological and social parameters, our ambition is to assess the inferential implications of key bio-psycho-social markers on MDT management, regarding PS, by introducing the concept of “Adapted Professional Activity” as a mirror of personalized “Adapted Physical Activity”.

### 4.1. Professional Inactivity in PSPS-T2 Patients: Psychological and/or Social Cause(s)?

The high prevalence rate of professional inactivity of PSPS-T2 patients found in this study (73.6%) is in accordance with the literature (50.9% to 81.5%) [14,15,16].

Three main hypotheses can be discussed: 

First, it has been suggested that professional inactivity can be related to remanent psychological distress [14,24,25,26,48,49,50]. Numerous studies have indeed reported a clear association between depression and professional status [14,51,52], in favour of a bidirectional influence of each parameter on the other, characterizing a genuine vicious circle. More specifically, unemployment has been reported to be correlated with psychiatric morbidity and elevated risk of suicide [53]. In a 4-year follow-up study, Bejerkeset et al. (2008) showed that unemployed persons were more susceptible to maintain their level of depression, whereas professionally active persons were more likely to decrease it [54]. With similar mental health before a cardiac event, Rost and Smith (1992) showed, after a 12-month period, that patients getting back to work after this medical event were less psychologically distressed compared to patients who remained unemployed at 12-month follow-up, despite no difference in mental health at baseline [55]. Our results corroborate all these findings, showing that inactive patients presented higher scores of depression, compared to active PSPS-T2 patients. 

Furthermore, this study highlights the fact that work-related kinesiophobia is greater in inactive than in active PSPS-T2 patients. All in all, we can clearly assume that psychological distress can be involved in difficulties to return to work and also that, above and beyond psychological distress, social factors need to be considered.

A second hypothesis stresses the potential role of the arduousness of the work related to social factors in patients with chronic pain [27,28,29,30,56]. In a survey addressed to 210 patients suffering from low back pain, Leclerc et al. (2009) showed that education level and SGH could be considered as risk factors for development of low back pain, especially in workers used to handling heavy loads [29]. In addition, Plouvier et al. [28] suggested that physical exposure at work was mainly involved in low back pain. They showed a negative correlation between SGH and time of exposure to biomechanical strain in 1487 professionals with persistent or recurrent low back pain. Hämmig and Bauer [56] showed, in a sample of 1846 professionals, a negative correlation between SGH and professional arduousness, indicating that low SGH patients were more likely to engaged in physical work. While 50% of the population with a low SGH reported that they regularly lifted heavy loads, this corresponded to less than 10% of the high-SGH population. In the same token, our study showed that inactive patients had a significantly lower SGH than active patients. Thus, social factors seem to have a high impact on functional capacity in chronic pain patients.

The influence of clinical parameters would constitute the third hypothesis to explain physical inactivity in PSPS-T2 patients. Indeed, it has been demonstrated in the literature that pain interacts with functional disability [57,58]. Static positions, prolonged standing or sitting (sedentary work), bending or twisting in extreme positions, exposure to heavy loading, forceful movements (e.g., pulling, pushing, etc.), vibrations are some of the many conditions that have been identified as causing pain and hampering return to work [59,60]. In parallel, it is commonly accepted that professional inactivity contributes to decreased physical health [61,62]. In our study, we showed that inactive PSPS-T2 patients presented higher levels of pain intensity and functional disability than active ones. Not surprisingly, the literature shows that the intensity of pain and reduced functional capacities negatively impacts quality of life of PSPS-T2 patients [8,22].

All in all, our results document that bio-psycho-social factors are involved in professional inactivity and deeply interlinked. While it clearly appears that biological, psychological and social factors need to be considered, the weight of each factor has still got to be determined. In a recent study using mixture models, Ounajim et al. [22] showed that PSPS-T2 patient profiles can be clustered in two classes. The first class, called “functional disability class”, corresponds to patients for whom health-related quality of life is mainly impacted by psychological factors and functional disability, whereas health-related quality of life of the second class, called “pain intensity class”, is dominated by the influence of psychological factors and pain intensity. The authors showed that health-related quality of life of male patients who perceived their jobs as “physically demanding” was more impacted by functional disability than by pain intensity. In agreement with this work, we assume that a new pain dimension should be integrated to multi-dimensional pain composite assessment [63], which will slightly change our practical approach, the objective being to propose an alternative way of work rehabilitation, along the PSPS-T2 patient pathway. Given the complexity of PSPS-T2 therapeutical approaches and, in contrast, given the need to transpose theoretical models to practical daily reality, inspiration should come from basic but robust solutions, taking country-related specificities into account.

### 4.2. The Problem Is Complex, the Solution Should Be Simple. The Concept of “Adapted Professional Activity” Inspired from the “Adapted Physical Activity” Model

Similarly to the notion of Physical Activity (PA), where PA has been identified to be responsible for injury but, a contrario, it has been found that physical inactivity may increase susceptibility to injury [64], it appears reasonable to assume that Professional Activity (ProA) may increase PSPS-T2 symptoms and that professional inactivity may increase the susceptibility of exacerbating PSPS-T2 symptoms. Travelling back to the 20th century, using DeLorean [65], practical guidelines for low pain management dictated cessation of all physical activities, to “protect and restore” body function. This idea now appears obsolescent since it has been strongly recommended to consider maintaining physical activity when in pain, and to adjust the level and the type of physical activity regarding a given patient’s specific capacities [66,67]. 

From the same perspective, ProA should be maintained with adjusted levels and demands, or by changing jobs and starting an adapted job related to the functional capability, defining the basis of the “Adapted Physical Activity” concept. While continuing to improve medical approach to treat PSPS-T2 patients, ProA could be considered as an important component of care pathway in these patients, notably regarding the employment protective effect [68,69]. It is indeed clearly established that work protects by anchoring the person in social life, giving him a sense of social usefulness and social respectability [68,69,70]. Even more, work protects by providing financial support to feed and care properly for themselves [70]. Thereby, Ragson et al. [71] have demonstrated among 64 blue-collar U.S. patients with end-stage renal disease patients that those who received social worker intervention were 2.8 times more likely to continue working than blue-collar who did not. This study suggests that it is social workers who are most likely to enable patients to maintain their professional activity [71]. The loss of work translates into a loss of self-esteem, causes isolation, loss of a network of help and essential social support [72]. In addition, it has been reported that professional inactivity contributes to a decrease in the mental and physical health of individuals [61,62]. Taken together, these findings suggest that “Adapted Professional Activity” should be considered as a therapeutic approach within the health promotion framework for unemployed PSPS-T2 patients [73], and could be proposed by extension in every chronic disability.

This “basic” conclusion could shock by its simplicity but in daily practice, PSPS-T2 patients, when assessed by MDT, systematically exhibit a high propensity to ruminate a potentially erroneous reprojection of their return to work, focused on a “black and white” vision: “Am I capable/or not?”. This construction might result from not only: (1) the clinical influence of their physical status on their abilities of introspection (including side effects of pain medications), (2) their fear of movement, sending them back to their “(un)usefulness” feeling/ psychologically altered self-representation, (3) low SGH profile, which would explain their limited capabilities of analysis, and/or social representations and/or subtle cognitive elaborations, BUT ALSO from: (1) inappropriate medical wording, occulting health literacy [74,75,76,77], (2) stereotyped medical management, focused on medications [78], in situations where personal coaching could be of significant influence [79] but is sorely lacking; and, (3) a frequently peremptory merciless verdict, to be digested in less than 5 min, regarding “professional capacity to return to work”, as assessed by a medical expert physician, leading to labelling “Return to work unacceptable”, this corresponds to social death from the patient’s perspective. 

In this context, reinforcement of the role of social workers and their integration in MDT pain structures would facilitate this process for two main reasons. First, as opposed to medical expertise, the opportunity for the social worker to dedicate sufficient time to the patient, given SGH-negative patient status and other factors of vulnerability, could constitute a crucial step toward analyzing previous work conditions and attempting to build a realistic professional project according to patient abilities. Second, by defining “potentiometers to adjust” with the patient, regarding not only physical parameters, but also psychological representations that might influence patient expectations and pain perception (to be elaborated through a psychological/psychiatric approach, as a psycho-social translational approach), the social worker would have the opportunity to put the patient back at the center. This social detonator could represent the first switch from a passive to an active patient position, which could then be transposed to ProA. 

This would require:

- personal coaching involving ergonomics/biomechanics, physiotherapy and occupational therapy approaches, tailored to each patient, to cultivate the concept of “Adapted Professional Activity”,
- a synergistic approach involving social workers, psychologists, focusing on patient interests,
- to insert social workers in pain structures and reinforce their integration into the MDT assessment and management, through therapeutic education.

Taking the liberty to place these considerations in historical perspective it bears mentioning that during the 19th century, people with physical or mental disability were not considered able to work [80,81], whereas today, even if it is not totally satisfactory, the social integration of disabled people has been the subject of major societal changes, such as improved work conditions designed to promote their social integration [82,83]. Historical perspective could explain that due to loss of self-esteem, social isolation aggravated by loss of financial resources, loss of a network of support care and essential social support in professional inactivity [83,84], the “philo-social” concept of enabling a patient to go back to work, even on a part-time basis, would help to ground this person back in social life and give him a sense of social usefulness and social respectability [68,69,70].

### 4.3. Strengths and Limitations 

Even though the proportion of inactive patients is in accordance with previous findings [13,14,15], it could be influenced by the organization of the French compensation system. Chaupain-Guillot and Guillot [85,86] have compared the impact of different European compensation systems on employee behavior. The authors showed that the absence of a waiting period and full salary maintenance significantly increases the probability of professional inactivity. It is therefore likely that our results can be transposed only to countries with a compensation system similar to France’s, as exists in the majority of the Organization for Economic Co-operation and Development member countries [86].

Second, in France, social workers can mobilize social assistance measures to help patients to maintain or regain their employment, such as free professional training or workstation adaptation of the financial component. The impact of social workers on professional situation could be conditioned by the existence of public policies, which vary between countries [87]. 

## 5. Conclusions

This prospective multicentric observational study underscores the fact that more than 2/3 of PSPS-T2 patients remain inactive despite Optimized Medical Management and that their working status does not change significantly after 1 year of follow-up. This study shows strong associations between unemployment and key clinical (EQ-5D-5L, NPRS scores) and psychological (HADS-D and FABQ-W scores) pain markers. 

In light of these study results, Professional Status should be considered by health professionals as a critical factor in the chronic pain management equation. Intense collaboration with social workers could facilitate the elaboration of a personalized professional plan, tailored to each patient and taking into account the influence of clinical, psychological and social parameters, through therapeutic education and prevention. 

This could ultimately guide intervention for this vulnerable population of PSPS-T2 patients, helping physicians to adopt the most appropriate temporal sequence in patient therapeutic pathway, with the initial goal of restoring “Adapted Physical Function” and the final objective of tailoring an “Adapted Professional Activity” matching with patient expectations and capabilities. 

## Figures and Tables

**Table 1:** Professional Status of patients in the PREDIBACK study.

Professional Situation	N	%
Active	40	26.5
Sick Leave	40	26.5
Disability	38	25.2
Long-term sick leave	16	10.6
Unemployed	17	11.3

**Table 2:** Correlation between professional situation and sociodemographic variables.

Variable	Active		Inactive		*p*-Value
	N	%	N	%	
**Age** (Mean; SD)	47.4	8.29	48.18	9.02	0.542
**Gender**					
Women	21	52.5	56	50.5	0.824
Men	19	47.5	55	49.5	
**Social Gradient of Health**					
Low	29	72.5	102	91.9	0.002
High	11	27.5	9	8.1	
**Education level**					
<upper secondary education	21	52.5	76	68.5	0.071
≥upper secondary education	19	47.5	35	31.5	

**Table 3:** Correlation between professional situation and medical assessment tools.

Variables	Active (*n* = 40)		Inactive (*n* = 111)		*p*-Value
	Mean	SD	Mean	SD	
**NPRS**	5.7	1.4	6.2	1.4	0.048
**ODI**	39.8	13.2	46.2	13.5	0.018
**EQ-5D-5L**	0.38	0.24	0.22	0.23	<0.001

NPRS: Numeric Pain Rating Scale; ODI: Oswestry Disability Index; EQ-5D-5L: EuroQol 5-Dimensional 5-Level.

**Table 4:** Comparison of psychological characteristics of patients according to professional situation.

Variable	Active (*n* = 40)		Inactive (*n* = 111)		*p*-Value
	Mean	SD	Mean	SD	
**FABQ-W**	10.6	9.2	21.7	9.0	<0.0001
**FABQ-PA**	15.2	6.5	16.6	6.7	0.225
**HADS-D**	7.5	3.4	9.3	4.2	0.019
**HADS-A**	9.5	3.8	10.6	4.1	0.117

FABQ-W: Fear-Avoidance Belief Questionnaire, Work subscale; FABQ-PA: Fear-Avoidance Belief Questionnaire, Physical Activity subscale. HAD-D: Hospital Anxiety and Depression Scale, Depression subscale; HAD-A: Hospital Anxiety and Depression Scale, Anxiety subscale; CSQ: Coping Strategies Questionnaire.

**Table 5:** Contingency table presenting professional situation evolution between the initial visit and the 12-month visit of the PREDIBACK study for patients of working age and normally active.

Professional Situation	INITIAL VISIT	12-Month Visit	*p*-Value
Unemployed	15	16	0.18
Active	25	32	
Sick Leave	30	19	
Disability and long-term sick leave	36	39	
Total	106	106	
